# Effectiveness of Psychological Counseling Intervention in High-Risk Pregnancies in Italy

**DOI:** 10.3390/jpm14090976

**Published:** 2024-09-15

**Authors:** Sofia Burgio, Gaspare Cucinella, Antonio Perino, Giovanni Baglio, Laura Crifasi, Robert Krysiak, Karolina Kowalcze, Giuseppe Gullo

**Affiliations:** 1 Department of Obstetrics and Gynaecology, Villa Sofia—Vincenzo Cervello Hospital, University of Palermo, 90128 Palermo, Italy; gaspare.cucinella@unipa.it (G.C.); antonio.perino@unipa.it (A.P.); l.crifasi@villasofia.it (L.C.); gullogiuseppe@libero.it (G.G.); 2 Research Unit, Italian National Agency for Regional Healthcare Services—AGENAS, 00187 Rome, Italy; baglio@agenas.it; 3 Department of Internal Medicine and Clinical Pharmacology, Medical University of Silesia, Medykow 18, 40-752 Katowice, Poland; rkrysiak@sum.edu.pl; 4 Department of Pediatrics in Bytom, Faculty of Health Sciences in Katowice, Medical University of Silesia, Stefana Batorego 15, 41-902 Bytom, Poland; kkowalcze@sum.edu.pl

**Keywords:** pregnancy, high-risk, counseling, psychological interventions, mothers, fathers

## Abstract

Background: The longitudinal study examines the effectiveness of a psychological support treatment for high-risk pregnancies using a between-groups design. It assesses the treatment’s impact on depression and fear of COVID-19 at three time points, and on prenatal attachment between the 20th and 24th weeks of gestation (T0), postnatal attachment 15–20 days after birth (T1), and three months after birth (T2). Additionally, the study evaluates the treatment’s effectiveness on PTSD related to childbirth and parental distress at T1 and T2. Methods: The study involved 117 parents experiencing high-risk pregnancies from a Sicilian hospital: 84 mothers (40 in the experimental group, 44 in the control group) and 33 fathers (19 in the experimental group, 14 in the control group). Results: ANOVA results showed that the psychological treatment was effective for maternal variables such as postnatal attachment and parental distress, and for paternal variables such as depression, prenatal attachment, PTSD symptoms, and parental distress (ANOVA, *p* < 0.05). Conclusions: The study highlights the growing evidence for providing continuous psychological support to couples with high-risk pregnancies, emphasizing that this support should extend beyond childbirth to assist families through this transition.

## 1. Introduction

The process of pregnancy, culminating in the birth of a child, stands as one of the most profound transformative experiences in an individual’s life. Although the common perception tends to idealize this journey as invariably positive and filled with joy, the literature in the field highlights that motherhood is often a period of change marked by anxiety, stress, disappointments, ambivalence, and frustrations—factors that society tends to overlook or deny [1,2].

Furthermore, consideration must also be given to the paternal perspective, which is often underestimated or disregarded. Unlike female postpartum depression, male postnatal depression has been the subject of fewer studies and has received less attention. However, a growing body of research suggests that men, particularly first-time fathers, can also experience mood disorders following the birth of a child [3,4,5].

Therefore, social support during pregnancy emerges as a critical resource and protective factor for the family, even in pregnancies classified as physiological—those that follow a typical, low-risk developmental course, free from pre-existing conditions or known risks to the mother and child.

In contrast, in cases of atypical development, where the pregnancy is classified as “high-risk” and is associated with complications or factors that may affect the health of the mother and fetus, psychological challenges—often unrecognized by society—can become particularly burdensome for the well-being of the child; both parents; and, consequently, the family as an institution, educational agency, and the child’s primary reference system.

These conditions constitute a genuine phenomenon of social fragility [5,6] that should not be underestimated, both in terms of enhancing research and interventions [7]. In such conditions, the development of parental competence can be severely compromised by the emotional stress, hyper-arousal, sense of uncertainty, and altered self-concept experienced by mothers and fathers [8]. 

Women experiencing high-risk pregnancies due to obstetric complications appear to have a notably higher likelihood of developing mental health disorders [9]. A recent review indicated that the prevalence of prenatal depression in this group ranges from 12.5% to 44.2% [10]. Numerous studies [11,12,13,14] have shown that an increase in maternal cortisol, as a physiological response to stressful conditions, appears to predict negative neonatal reactivity and influence the infant’s temperament in addition to causing a psychological vulnerability during the perinatal period, which, when combined with obstetric complications often associated with high-risk pregnancies, can lead to postnatal symptoms indicative of post-traumatic stress disorder (PTSD) [15].

Moreover, maternal stress during pregnancy can reduce maternal immune defenses and, consequently, those of the child [16,17,18]. The increased stress experienced during high-risk pregnancies may affect the development of prenatal attachment [19], and it is well established that the mother–child relationship in the postpartum period is strongly correlated with prenatal attachment [20,21]. As is known, maternal responsiveness to the child’s needs encompasses behavioral, communicative, and emotional aspects and reflects the mother’s ability to share both the positive and negative emotions of the child. The parent’s ability to attune to the child’s emotions serves as an indicator of the type of attachment the child will develop [22].

In this context, the study addressed the social need to ensure and protect children’s developmental health, considering the short- and long-term effects of the prenatal environment. Indeed, infants of mothers with high levels of stress during pregnancy are not only at greater risk of preterm birth, low birth weight, and organ prematurity, but may also suffer significant negative effects on neurobehavioral organization in the long term (cognitive and behavioral problems, risk of ADHD [9,23,24], anxiety, and language development problems) [25].

Further consideration should be given to the negative emotional impact that the COVID-19 pandemic may have had on pregnant women and their partners, causing fear and stress. Negative emotions during pregnancy can subsequently influence the fear of childbirth and may also have consequences in the postpartum period [26].

It thus appeared interesting to evaluate the effectiveness of a specific support program on maternal and paternal variables from prenatal to neonatal stages, such as the presence or absence of depression, prenatal and postnatal attachment, the presence of possible post-traumatic stress disorder related to labor and delivery, and consequent difficulty in managing their parental role, through parental distress indices.

Here, reference is made to the transformative value of a specific counseling service aimed at prevention and the possibility that it can genuinely promote the development of strategies and skills necessary to navigate risk and contribute to the creation of a theoretical framework in the research area [9]. Specifically, the counseling aims to address the entire psychological complexity of the parents in the here and now of their condition, engaging in a process of redefining meanings and personal constructs [27]. On the one hand, the importance of establishing a helping relationship with the parent, which can support their personal journey through risk by developing coping skills and resources [28], was highlighted. On the other hand, the need to work on the constructs that guide parental competence was emphasized.

This helping relationship acts as a mediating function [29] between the different temporal phases experienced by the parent (e.g., the succession of different weeks of pregnancy, the time needed to adapt to the new condition, personal life cycle time, family life cycle time) and between different spatial contexts (such as hospital spaces, everyday life, and mental spaces related to the integration of new and old self-representations); moreover, it mediates between new and old representations of oneself and others and acts as an activation of links between different contexts.

Additionally, this relationship, through a protective function [30], facilitates the activation of constructs related to self-esteem and self-efficacy, the ability to manage emotions, and problem-solving, promoting the formation of new representations of the parental role in terms of competence. Another fundamental aspect of the helping relationship is social support [31], which translates into cognitive support through the availability of information and clarification of key concepts for the parent, as well as emotional support, which activates listening and containment of emotions.

Among the functions of this counseling are also empowerment [32], which aims to make the parent a conscious and responsible subject within their situation, and assessment [33], which focuses on analyzing the complexity of their psychological state. The psychological intervention model employed during the counseling sessions incorporated several fundamental elements [34], i.e., listening [35], accompaniment [36], reception [37], mentoring, event anticipation [38], and adaptation [39].

Each intervention prioritized listening, utilizing specific detection techniques to enable mothers and fathers to explore alternative approaches to managing events and emotions. Within this framework, accompaniment provided the opportunity for “joint understanding”, while mentoring facilitated the identification and implementation of the proposed alternatives. A distinguishing feature of this model was its focus on “event anticipation” [38]. By addressing dysfunctional catastrophic anticipations, it was possible to guide a restructuring of these anticipations through social support.

The model also integrated social support [40] and its buffering effects [41] alongside psychological, educational, and psychosocial rehabilitation interventions [42,43], and employed clinical, observational, experiential, and psychodiagnostic methodologies.

It is also necessary to distinguish direct counseling from telephone counseling provided during the support intervention; the former aimed to activate a transformative helping relationship functional, as already mentioned, to the redefinition of personal constructs [44], unlike telephone counseling, which aimed to activate a supportive relationship aimed at developing the mentalization of change.

This distinction is necessary because it carries the definition of different specific spaces and times for the two types of counseling: direct counseling took place within the outpatient setting, thus also following what was immediately activated concerning the gynecological visit; telephone counseling was activated at a later time, outside the hospital context.

In light of what has been presented, the following general objectives of the research pathway are identified:

- Verify the effectiveness of the counseling intervention lasting nine sessions (50 min per session, three sessions for each phase of the research) on the variables.

Specifically, the effectiveness of the treatment on depression and fear of COVID-19 at the three phases is investigated, as well as prenatal attachment between the 20th and 24th weeks of gestation (T0) and postnatal attachment 15/20 days after birth (T1) and at three months of the child’s life (T2). The effectiveness of the intervention on the presence of possible PTSD related to the birth experience and parental distress at T1 and T2 is evaluated. In this sense, these variables were measured only in the last two phases of the research, precisely because they are related to the concrete experience of childbirth and being a parent.

In light of these objectives, the following hypotheses are formulated:

- It is hypothesized that the treatment will significantly activate, compared to the control group, a reduction in levels of depressive symptoms and fear of COVID-19 both during pregnancy and the postpartum period. Additionally, it is hypothesized that it will reduce the presence of possible PTSD and stress related to the maternal and paternal roles during the postpartum period.
- It is hypothesized that the treatment will significantly activate, compared to the control group, an increase in the indices of prenatal attachment and postnatal attachment.

## 2. Materials and Methods

From a methodological perspective, the study was defined through a longitudinal and between-groups design (experimental group and control group). Specifically, an evaluation was planned of the possible well-being outcomes promoted by the support program described below, aimed at mothers and fathers from prenatal to neonatal periods, who will constitute the experimental group, through the measurement of the study variables: depression, fear of COVID-19, prenatal attachment, postnatal attachment, presence of possible post-traumatic stress disorder (PTSD) related to the birth experience, and parental distress.

### 2.1. Participants

A total of 117 parents experiencing high-risk pregnancies (N = 117) were recruited from a High-Risk Pregnancy Clinic at a Sicilian hospital: 84 mothers, with 40 assigned to the experimental group and 44 to the control group, and 33 fathers, with 19 assigned to the experimental group and 14 to the control group (Table 1).

The inclusion criteria were having received a diagnosis of high-risk pregnancy and understanding the Italian language for the administration of psychometric tests.

The target population consisted of pregnant women aged 18 to 45 residing in urban areas. A simple random sampling method was used, and to ensure sample randomization, a sampling frame (i.e., a list randomly generated by the gynecological appointment booking center) was utilized. Through the random selection method, individuals were chosen from the list by drawing lots. Each individual had an equal probability of being selected, ensuring that the process was fair and unbiased.

### 2.2. Procedure and Tools

The sample was recruited during the gynecological visit, where the woman and, subsequently, her partner were invited to participate in the research project and were provided with and read the informed consent to the study. Participation was voluntary and anonymous, and the time required to administer the psychometric instruments was approximately 20 min.

The research project included the use of the following measures:

- A socio-demographic sheet specifically created for this study, collecting data on gender, age, nationality, couple status, educational level, type of occupation, presence of pathologies, fetal issues, and presence or absence of miscarriage threats.
- Beck Depression Inventory II (BDI) [45] is a self-report questionnaire consisting of 21 items, aimed at measuring cognitive, motivational, affective, and behavioral symptoms of depression. Each item is scored from 0 to 3, and the higher the BDI score, the greater the level of depression. Possible item examples include: “I feel sad most of the time”, “I always feel sad”, or “I feel so sad or unhappy that I can’t stand it”. Regarding psychometric properties, the instrument shows good internal consistency and reliability with Cronbach’s alpha values (α = 0.90).
- Fear of COVID-19 (FCV-19S) [46] is a 7-item scale that assesses the fear of COVID-19. The seven items are rated on a 5-point Likert scale from 1 (strongly disagree) to 5 (strongly agree), with scores ranging from 7 to 35. The higher the score, the greater the fear of COVID-19. Possible item examples are: “I am very afraid of COVID-19” or “I cannot sleep because I worry about catching (or having) COVID-19”. The FCV-19S also shows good internal consistency and reliability (α = 0.84).
- Parenting Stress Index—Short Form (PSI) [47]: a self-report questionnaire that assesses the level of distress perceived in relation to one’s parenting role and the parent-child relationship, with 36 items. It consists of three subscales: parental distress (PD), parent–child dysfunctional interaction (PCDI), and difficult child (DC). The sum of these subscales provides a total stress score. Additionally, the PSI-SF includes a defensive responding scale (DIF). In this study, only the total stress score was used. Possible item examples include: “I often feel that I am not handling things well” or “To meet my child’s needs, I find myself sacrificing my life more than I expected”. The instrument shows good internal consistency and reliability for its psychometric properties (α = 0.78).
- Impact of Event Scale-Revised (IES-R) [48]: a self-report measure to assess symptomatic responses to specific traumatic stressors in the first seven days after exposure to the traumatic event, in this case, after childbirth. It consists of 22 items measuring symptoms of hyperarousal, intrusion, and avoidance. In this study, only the total score of the scale was used. Possible item examples are: “Anything that reminded me of it made me feel emotions related to it” or “Other things kept making me think about it”. The instrument shows good internal consistency and reliability for its psychometric properties (α = 0.73).
- Prenatal Attachment Inventory (PAI) [49]: a self-report questionnaire with 21 items that investigates maternal–fetal attachment as a single dimension. It is used to measure the attachment levels of pregnant women to their unborn babies. Possible item examples are: “I wonder what the baby is like now” or “I imagine calling the baby by name”. The instrument shows good internal consistency and reliability for its psychometric properties (α = 0.75).
- Paternal Antenatal Attachment Scale (PAAS) [50]: a self-report questionnaire on prenatal attachment (16 items) that focuses on two dimensions: the quality and intensity of concerns related to prenatal attachment. The first subscale measures the quality of the parent’s affective experience towards the unborn child (e.g., feelings of tenderness vs. feelings of detachment or irritation). The second subscale measures the intensity of feelings towards the fetus and the amount of time spent thinking or worrying about the baby. The PAAS also shows good internal consistency and reliability (α = 0.71).
- Postpartum Bonding Questionnaire (PPBQ) [51]: This tool is designed to identify perceived disturbances in the mother-child relationship (25 item). It consists of 4 scales:
  Scale 1 (Impaired Attachment): Provides a general factor for identifying certain types of mother–child bonding disorders.
  Scale 2 (Rejection/Anger): Detects the presence of severe mother–child relationship disturbances.
  Scale 3 (Anxiety about Care): Used to identify anxiety focused on the child.
  Scale 4 (Risk of Abuse): Identifies the risk of abuse.

In this study, only the general factor for identifying certain attachment issues was considered, specifically Scale 1. Examples of items include “I feel close to my baby” or “I wish the old days when I had no children would return”. Regarding psychometric properties, the PPBQ also shows good internal consistency and reliability (α = 0.71).

### 2.3. Treatment

**Figure 1:**
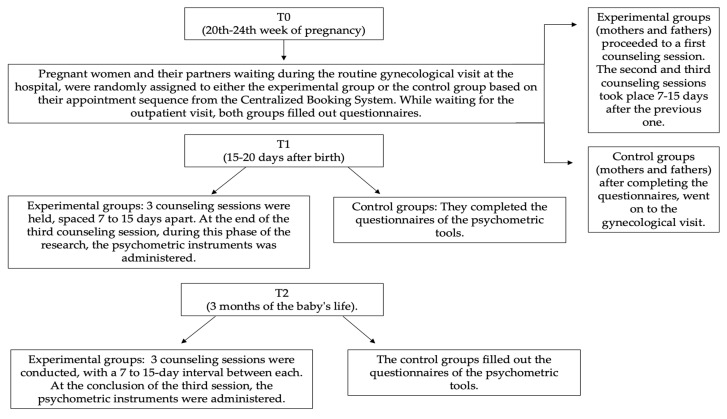
Flow chart of counseling process.

The research was structured into three phases: T0 (20th–24th week of pregnancy), T1 (15–20 days after birth), and T2 (3 months of the baby’s life) (see Figure 1). In each phase, a supportive treatment consisting of 3 counseling sessions was applied, and the outcomes were measured using a comprehensive battery of psychological instruments.

Specifically, as emphasized by the theoretical framework of reference, the counseling aimed to manage the emotional and psychological complexity of the parents by addressing their situations in the here and now. This process involved reworking their personal meanings and beliefs [52]. It was important to build a supportive relationship with the parents to help them identify their resources, strengthening their skills and coping strategies [29]. Additionally, it was necessary to address the mental constructs that influence their parenting skills. The sessions focused on mediation, protection, social support, and empowerment functions, guiding each topic addressed throughout the process.

Within a High-Risk Pregnancy Clinic at a Sicilian hospital, pregnant women between the 20th and 24th week of gestation were randomly assigned to either the experimental group or the control group based on their appointment order from the Centralized Booking System, following a randomized control trial perspective.

During the wait for the outpatient visit, both groups completed questionnaires presented in booklet form. Women assigned to the experimental group then proceeded to a first counseling session, while those in the control group, after completing the questionnaires, went on to the gynecological visit. The same procedure was applied to the men in the waiting room; if not present, they were contacted by phone.

For the experimental group, two additional counseling sessions were conducted at intervals of one to two weeks. Phase T2 of the research started within 15–20 days after birth, and at the end of the last of the three counseling sessions, the booklet of psychometric instruments was administered.

Similarly, in T3 of the research, around 3 months of the baby’s life, three counseling sessions were conducted, and at the end of the third session, the psychometric instrument booklet was completed.

The control group followed the same timeline, only completing the psychometric instruments without receiving the counseling sessions.

### 2.4. Data Analysis

The *t*-test was calculated for the only continuous variable present in the study, namely age. The *t*-test values are reported in Table 2.

Preliminary analyses (means, standard deviations, and percentages) were conducted for the socio-demographic variables (Table 3). 

Means and standard deviations were calculated for the study variables (depression, fear of COVID-19, prenatal attachment, postnatal attachment, presence of post-traumatic stress disorder related to childbirth, and parental distress).

A regression analysis was conducted to verify the pre-homogeneity test between the experimental group and the control group for each variable.

An independent-samples *t*-test was conducted for the variables related to maternal prenatal attachment (PAI); similarly, a *t*-test was conducted for paternal prenatal attachment (PAAS).

For both mothers and fathers, a repeated-measures ANOVA design 3 (T0, T1, T2) × 2 (experimental group and control group) was adopted for the variables depression (BDI) and fear of COVID-19 (FCV-19S).

A 2 (T1, T2) × 2 (experimental group and control group) design was used for the variables postnatal attachment (PPBQ), presence or absence of PTSD (IES-R), and parental distress (PSI).

## 3. Results

The average age of the women in the experimental group was 30.0 years (SD = 6.51), while that of the men was 30.7 years (SD = 5.34); in the control group, the average age of the mothers was 29.7 years (SD = 5.64), while that of the men was 35.3 years (SD = 8.56) (Table 2). Most of the women were Italian (97.6%), as were the men (97%). The most common couple status for both parents was marriage (60.7%).

Most of the women had already had two children before the current pregnancy (29.68%), while the group of fathers was mostly composed of men experiencing fatherhood for the first time (33.3%); specifically, 20.2% of the mothers in the experimental group were first-time mothers, the same number as women already in their third pregnancy (20.2%). Most of the men in the experimental group had not yet had fatherhood experiences (24.2%) compared to those in the control group, who declared they already had two children (15.2%).

Regarding the education level, both women (56.6%) and men (42.4%) had lower-secondary-school diplomas. A total of 67.9% of the women were housewives, while the most common occupation among the men was laborer. The most common pathological condition among the women was diabetes, both in the experimental group (50.2%) and in the control group (48.1%); despite this, most of the women declared that they had no particular health problems for the fetus during pregnancy (62.5% in the experimental group and 77.3% in the control group), nor were they experiencing a threat of miscarriage (67.5% in the experimental group and 90.9% in the control group) (see Table 1).

Homogeneity checks were conducted for the demographic characteristics of the experimental and control groups, as well as for the pre-measurements (T0) of each effect variable. The effectiveness of randomization was assessed by comparing the groups using the most appropriate tests for each type of variable (*t*-test, Chi-square).

Regarding the comparison between groups based on maternal age, there were no significant differences (*p* = 0.81), nor were there significant differences for nationality (*p* = 0.17), relationship status (*p* = 0.61), education level (*p* = 0.68), employment type (*p* = 0.16), medical condition (*p* = 0.95), or the presence of fetal problems (*p* = 0.13). However, a significant difference was found between the groups for the maternal variable “threat of miscarriage” (*p* = 0.008).

As for paternal age, a significant difference was observed at the 5% level (*p* = 0.04). No significant differences were found between the groups concerning paternal nationality (*p* = 0.23), number of children (*p* = 0.65), education level (*p* = 0.09), or employment type (*p* = 0.14).

Overall, randomization was successful for most variables, except for the paternal age variable (*p* = 0.04) and the threat of miscarriage for mothers (*p* = 0.008).

The regression analysis was conducted to assess the correlation of factors that had been found to be unbalanced between the two groups, demonstrating that in no case was there a correlation between these factors and the outcomes studied, thereby eliminating the possible existence of confounding factors.

Since a confounding factor must correlate with both the outcomes and the treatment, if it only covaries with one, it is not a confounding factor.

The regression model, in fact, showed no correlation between the threat of miscarriage and impaired bonding at T1 (*t* = 0.98, *p* = 0.33) and at T2 (*t* = 0.11, *p* = 0.91).

In the same regression model, the treatment was included and remained significant for all outcomes (*t* = −3.00, *p* = 0.004), while the threat of miscarriage was not significant (*t* = 0.11, *p* = 0.91).

Similarly, for paternal variables, the regression model showed that there was no significant correlation between BDI and age at T1 (*t* = −0.87, *p* = 0.39) or at T2 (*t* = −0.86, *p* = 0.39). When the treatment was included in the same regression model, it remained significant (*t* = 2.69, *p* = 0.012), while age showed no significance (*t* = −0.09, *p* = 0.92).

The regression model did not show significant correlations between IES-R and age at T1 (*t* = 0.03, *p* = 0.98) or at T2 (*t* = −0.16, *p* = 0.87). When the treatment was included in the same regression model, it remained significant (*t* = 3.78, *p* = 0.001), while age showed no significance (*t* = 0.95, *p* = 0.34).

Finally, the regression model showed no significant correlation between PSI and age at T1 (*t* = −1.04, *p* = 0.30) and at T2 (*t* = −1.59, *p* = 0.12). When the treatment was included in the same regression model, it remained significant (*t* = 6.71, *p* < 0.001), while age showed no significance (*t* = −0.34, *p* = 0.73).

Descriptive statistics are presented in Table 1.

The means and standard deviations for the study variables are reported in Table 3 for mothers and in Table 4 for fathers.

The *t*-test showed that there were no significant differences regarding maternal prenatal attachment between the experimental group and the control group [*t* (1, 84) = −1.64, *p* = 0.10].

Conversely, the *t*-test showed significant differences between the experimental group and the control group for the variable paternal prenatal attachment [*t* (1, 31) = 2.23, *p* = 0.03]. In this regard, the means indicate that the experimental group had a higher PAAS score (M = 64.0, SD = 6.66) compared to the control group (M = 58.4, SD = 7.62).

Regarding the data related to mothers, the repeated-measures ANOVA highlighted that, for the variable depression (BDI) between T0, T1, and T2, there were significant differences within subjects in the interaction between BDI and group [F (2, 82) = 25.4, *p* < 0.001, η^2^ *p* = 0.237]. The data analysis also showed how the mean BDI scores changed over time, indicating a decrease in scores across the three time points (M = 6.64, SD = 5.64 at T0; M = 5.01, SD = 4.64 at T1; M = 4.96, SD = 4 at T2). No significant differences were found between groups.

The interaction effect between the fear of COVID-19 variable and group across the three research time points was not significant, and there were also no significant differences between groups.

**Figure 2:**
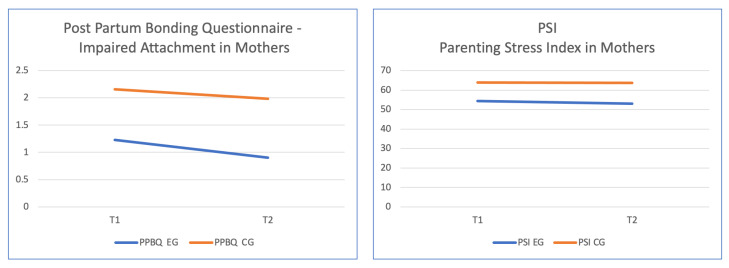
Significant differences between means for the variables compromised attachment (PPBQ) and parental distress (PSI) in mothers from the experimental group and the control group.

Regarding the variable impaired bonding, there were no significant differences within subjects in the interaction between PPBQ and group, but significant differences were found between groups [F (1, 83) = 7.54, *p* = 0.007, η^2^ *p* = 0.084]. Data analysis showed how the mean scores on PPBQ decreased for both groups, with scores being lower in the experimental group (M = 1.23, SD = 1.51 at T1; M = 0.90, SD = 1.03 at T2) compared to those in the control group (M = 2.16, SD = 2.21 at T1; M = 1.98, SD = 1.90 at T2). Figure 2 displays the mean scores for the PPBQ variable in the two groups.

The interaction effect between the parenting stress (PSI) variable and group between T1 and T2 did not show significant differences, whereas significant differences between groups were found [F (1, 83) = 64.1, *p* < 0.001, η^2^ *p* = 0.439]. Data analysis revealed how the mean scores on PSI decreased for both groups, with scores being lower in the experimental group (M = 54.3, SD = 8.56 at T1; M = 53.1, SD = 6.14 at T2) compared to those in the control group (M = 63.9, SD = 4.32 at T1; M = 63.7, SD = 4.44 at T2).

For the variable presence of PTSD (IES-R), between the last two time points of the study, significant differences within subjects were evident [F (1, 82) = 8.49, *p* = 0.005, η^2^ *p* = 0.094], but there were no significant differences between groups. Data analysis also showed how the mean scores on IES-R changed over time, indicating an increase in scores across the two time points (M = 0.27, SD = 0.47 at T1; M = 0.34, SD= 0.61 at T2).

Regarding fathers, the repeated-measures ANOVA highlighted significant differences within subjects among T0, T1, and T2 for the BDI variable in the interaction between BDI and group [F (2, 31) = 6.66, *p* = 0.002, η^2^ *p* = 0.177], as well as significant differences between groups [F (2, 31) = 4.92, *p* = 0.034, η^2^ *p* = 0.137]. Data analysis showed that the mean BDI scores decreased in the experimental group over the three study periods (M = 3.26, SD = 3.19 at T0; M = 3.05, SD = 2.90 at T1; M = 2.79, SD = 2.66 at T2) compared to the control group, where they increased (M = 5.71, SD = 5.66 at T0; M = 6.57, SD = 6.66 at T1; M = 7.29, SD = 6.06 at T2).

The interaction effect between the fear of COVID-19 variable and group across the three research periods showed significant differences [F (2, 31) = 3.94, *p* = 0.024, η^2^ *p* = 0.113], but there were no significant differences between groups. Data analysis showed that the mean scores on the FCV-19S changed over time, indicating a decrease in scores across the three periods (M = 14.9, SD = 6.77 at T0; M = 17.4, SD = 8.65 at T1; M = 14.5, SD = 5.46 at T2).

**Figure 3:**
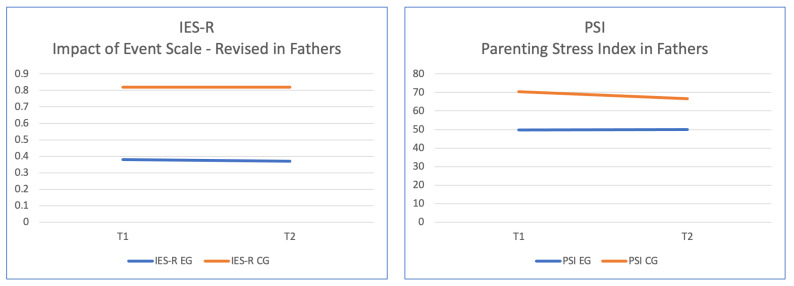
Significant differences between means for the variable presence of post-traumatic stress disorder (IES-R) and parenting distress (PSI) in fathers of the experimental group and the control group.

The interaction effect between the parenting stress index (PSI) variable and group between T1 and T2 showed significant differences [F (1, 32) = 6.60, *p* = 0.015, η^2^ *p* = 0.176], as well as significant differences between groups [F (1, 32) = 74.5, *p* < 0.001, η^2^ *p* = 0.706]. Data analysis showed that the mean scores on the PSI were lower for the experimental group; however, the scores over time seemed to slightly increase (M = 49.7, SD = 7.92 at T1; M = 50.1, SD = 8.22 at T2) compared to the control group, where scores decreased (M = 70.3, SD = 3.58 at T1; M = 66.6, SD = 2.73 at T2), but the control group still reported higher scores than the group that received the treatment (Figure 3).

The interaction effect between the variable presence of PTSD (IES-R) and group at T1 and T2 did not show significant differences, while significant differences were found between groups [F (1, 32) = 11.9, *p* = 0.002, η^2^ *p* = 0.278]. Data analysis showed that the mean scores on IES-R decreased for both groups, with the scores being consistently lower in the experimental group (M = 0.39, SD = 0.42 at T1; M = 0.37, SD = 0.41 at T2) compared to those in the control group (M = 0.82, SD = 0.44 at T1; M = 0.82, SD = 0.28 at T2).

## 4. Discussion

The results of the study highlighted and evaluated the potential well-being outcomes promoted by the described support pathway, from the prenatal to neonatal stages. 

The data obtained, in general, lead to the hypothesis that, in conditions of high-risk pregnancy, psychological counseling is an effective intervention for detecting changes in the variables under study, particularly for fathers. 

Regarding the research hypothesis concerning improvement in prenatal attachment levels, the results show a statistically significant difference in paternal prenatal attachment between the experimental and control groups. 

The difference in mean scores on the fathers’ questionnaires showed higher scores in the experimental group. This finding aligns with sector literature, as some studies emphasize that the quality of paternal prenatal attachment is higher when fathers experience fewer depression symptoms during pregnancy, are young, and are expecting their first child [53,54].

In this regard, both the low BDI scores obtained by the experimental group across all three study times and the descriptive statistics of the sample, which indicate that the majority of fathers in the experimental group were first-time fathers (24.2%), support the presence of better-quality prenatal attachment.

The analysis also revealed significant differences in terms of decreasing scores over the three study times regarding depression indices for mothers, although there were no significant differences between the experimental and control groups. 

This finding contrasts with some studies in the literature [55,56], which instead support prenatal to neonatal counseling intervention as effective in improving depressive symptoms. It should be noted, however, that the mean scores on the women’s questionnaires across all three study times did not indicate the presence of clinically significant mood disorders in either the experimental or control groups (BDI < 10).

As indicated by the World Health Organization (WHO), antenatal care (ANC) coverage is a critical indicator of access to and utilization of healthcare services during pregnancy [57]. Receiving antenatal care at least four times increases the likelihood of receiving effective maternal health interventions during the prenatal period. Perinatal depression (PND) conditions are common complications that occur during pregnancy or within the first year postpartum; because these conditions often go unrecognized and have devastating effects on mothers and children [58], perinatal health care programs should always monitor depression indices. Specifically, the fact that the sample of mothers examined did not report clinically significant depression indices, despite a change over time in terms of reduced depression levels, may suggest that receiving various forms of antenatal care at least four times, as recommended by WHO, had a positive impact on these women’s mental health. As highlighted by Srisurapanont et al. [58], it may be interesting to measure these depression indices using other ANC screening tools that are more widely used in different countries, considering the concept of (functional) disability, which differentiates from non-disability by evaluating whether individuals face significant limitations in their capacity to carry out daily activities and tasks or if they are functioning as expected.

No statistically significant difference was found between the interaction effect of the fear of COVID-19 variable and the group over the three study times, nor was any difference found between the experimental and control groups. 

These data are intriguing, especially considering that the average scores for both groups of women did not indicate significant COVID-19 fear. This contrasts with studies in the field [59,60] which have emphasized how mothers experiencing high-risk pregnancies have felt negative emotions such as fear, worry, stress, and anxiety about COVID-19. 

Specifically, living through a high-risk pregnancy condition caused concern among women who imagined it would expose them and their fetus to a greater risk of harm and danger [61].

Postnatal attachment in mothers did not show statistically significant differences in the last two study time points. Although some studies have emphasized how mother–child attachment significantly grows over time [62,63], the development of prenatal attachment relationships does not necessarily follow a direct and linear path. The evidence in studies is not uniform, and several investigations have detected only a moderate link, for example, between attachment before and after birth, indicating that the attachment that develops after birth is not widely determined by prenatal attachment [64].

However, data analysis showed statistically significant differences regarding the experimental group and control group; thus, the counseling intervention supported a change in the experimental group, improving the mother–child relationship as indicated by industry literature [65,66].

Furthermore, the results did not show significant differences regarding the parental distress variable between T1 and T2, underlining how this finding is inconsistent with studies in the literature; specifically, this research reveals how the transition from birth to the first months of a child’s life may be a vulnerable time for mothers, during either premature birth [45] or normal term pregnancies [67].

Also, for the parental distress variable, significant differences were found between the experimental group and the control group, with lower scores on the questionnaires for the experimental group, indicating that the counseling intervention was effective in reducing the stress of mothers, as is supported by some studies in the field [68,69].

Regarding the presence of PTSD, the results showed significant differences at T1 and T2 in women, indicating a change from immediately postpartum to three months of a child’s life, although there were no significant differences between the experimental and control groups. The averages show that such a change forecasted an increase in both groups, and this result is supported by industry literature; indeed, birth experience can be a particularly traumatic event, leading to the development of PTSD symptoms over time [70,71]. Moreover, there are several studies in the literature emphasizing how variables related to childbirth, maternal health, and neonatal health in high-risk pregnancies might be compromised, and this might have an effect on PTSD symptoms [72,73].

Regarding the results reported by fathers, there were significant differences in the three research times for the depression variable, as well as significant differences between the experimental group and the control group. Specifically, the results showed a change in scores over time in terms of decreasing depression levels for fathers in the experimental group and increasing scores for questionnaires on depressive symptomatology in fathers in the control group. Although the average scores indicate the absence of clinically significant mood disorders (BDI < 10), international scientific studies demonstrate that paternal depressive symptomatology is always higher during pregnancy and the postpartum period [74,75].

The psychological intervention was shown to be effective, as supported by some studies in the field [76,77], although there are still no guidelines that can guide clinical intervention with fathers from prenatal to neonatal stages. 

Regarding the fear of COVID-19 variable, fathers showed a significant change over time, but no significant difference regarding the experimental and control groups. The fear of COVID-19 in the paternal sample from prenatal to neonatal is not a parameter found in the industry studies; some studies have focused on the fear of COVID-19 in the relationship of interactions with childbirth, emphasizing the direct influence that this variable has not only on the fear of paternal birth, but also on maternal birth [78]. However, information is not provided on the change in this variable over time in relation to the pregnancy, childbirth, and home transition in the first months of a child’s life.

The results still show significant differences between the parental distress variable and the group at T1 and T2, as well as significant differences between the experimental and control groups. This data are interesting in that, although the average score of the experimental group was lower than that reported by the control group, parental distress seemed to increase in the experimental group from birth to three months of a child’s life compared to the group that did not receive treatment. 

This could be attributed to the fact that the psychological counseling conducted with these fathers encouraged them to reflect on and focus on the challenges of being a parent to a newborn from a high-risk pregnancy, where paternal difficulty often lies in addressing negative emotions and critical aspects of the parenting experience [79]. Fathers often tend to prioritize what concretely contributes to the well-being of the baby and the mother, thereby sometimes neglecting and choosing to “ignore” the more critical emotional aspects. Asking these fathers to instead focus on these critical aspects may have led to a greater perception of parental distress in managing the newborn.

Lastly, the results did not show statistically significant differences between the presence of possible PTSD and the group at T1 and T2. However, significant differences were found between the experimental and control groups, indicating a slight improvement in the group that received counseling intervention.

Despite some studies showing symptoms attributable to PTSD in fathers after childbirth, no literature exploring aspects of PTSD intervention in fathers has been found, except for some therapeutic suggestions from the examined studies [80].

These suggestions underscore the importance of providing fathers with a supportive space from prenatal to neonatal stages which is specifically designed to support them in managing emotions and postnatal care [3], as intended by the counseling intervention proposed.

Regarding the clinical implications, the study highlights the importance of psychological support interventions from the prenatal to the neonatal period, especially in low- and middle-income settings, such as the reference sample. Pokharel et al. [81] emphasize how common perinatal mental disorders are prevalent among parents in low- and middle-income countries. These non-psychotic mental health conditions, such as depression and anxiety, negatively impact daily life and, if left untreated, are associated with adverse obstetric and neonatal outcomes, such as miscarriages and stillbirths, which in turn can further worsen the parents’ mental health. 

The importance of psychological support [82] from the prenatal to the neonatal period can benefit in the future from innovative approaches such as tele-counseling, which can provide necessary support during the transition to parenthood, even to the less affluent segments of the population [83].

As suggested by Jatchavala et al. [84], global perinatal mental health policies should always reflect the integration of evidence-based research and standardized practices. Only through the combination of experimental research and clinical practice can we achieve global guidelines for parental care and, consequently, child development.

## 5. Conclusions

The proposed study supported the increasing evidence-based necessity of providing continuous psychological support to women and men experiencing a high-risk pregnancy. 

In particular, the psychological support intervention was effective in improving postnatal attachment between mother and child and reducing parental distress experienced by mothers, making them more confident and competent in managing difficulties. 

Regarding the effectiveness of the intervention on paternal variables, psychological counseling proved supportive in reducing depression levels, improving prenatal attachment, and decreasing the perception of PTSD-related symptoms.

In light of the results obtained, the support should not end at the birth of the baby but should accompany the couple, and thus the family, through this delicate developmental transition. The opportunity to be supported and to have an exclusive listening space can enable them to feel more competent in managing their parental role by identifying and enhancing their specific resources.

One limitation of the study is certainly the small number of participants, especially fathers, and the lack of opportunities to make the support intervention more comprehensive through experiential and laboratory methods. These methods were impossible to implement due to the restrictions imposed by the COVID-19 pandemic.

## Figures and Tables

**Table 1:** Descriptive statistics of the sample (N = 117).

Variables	Women (N = 84)				Men (N = 33)			
	Mean		SD		Mean		SD	
	EG	CG	EG	CG	EG	CG	EG	CG
Age	30	29.7	6.51	5.64	30.7	35.3	5.34	8.56
Nationality								
*Italian*	97.50%				97%			
*Foreign*	2.40%				3%			
Couple’s Conditions:								
*Married*	60.70%				60%			
*Cohabiting*	38.10%				40%			
*Single*	1.20%							
Number of children beyond pregnancy								
*0*	28.60%				33.30%			
*1*	27.40%				15.20%			
*2*	29.80%				24.20%			
*3*	7.10%				12.10%			
*>3*	7.10%				15.20%			
Level of Education:								
*Primary School*	4.80%							
*Middle School*	56%				9.10%			
*Professional School*	19%				42.40%			
*High School*	15.50%				30.30%			
*Degree*	3.60%				15.20%			
*Phd/Specialization*	1.20%				3%			
Job Condition:								
*Housewives*								
*Student*	67.90%							
*Workmen*	2.40%				57.60%			
*Employee*	3.60%				18.20%			
*Trader*	19%				3%			
*Free Lance*					6.10%			
*Unemployed*	1.20%				12.10%			
*Looking for first job*	6%				3%			
Pathologies	EG		CG					
*Diabetes Mellitus*	50.20%		48.10%					
*Obesity*	22.30%		25.30%					
*Hyperthension*	14.40%		15.40%					
*Cardiac pathologies*	11.10%		10.20%					
*Autoimmune diseases*	2%		1%					
Fetal problems								
*Yes*	37.50%		22.70%					
*No*	62.50%		77.30%					
Threat of abortion								
*Yes*	32.50%		9.10%					
*No*	67.50%		90.90%					

**Table 2:** *t*-test for age variable.

Variables	Mothers					Fathers				
	E G	C G	*t*	df	*p*-Value	E G	C G	*t*	df	*p*-Value
	M (SD)	M (SD)				M (SD)	M (SD)			
**Age**	30 (6.51)	29.7 (5.63)	−0.23	82	0.81	30.7 (5.34)	35.3 (8.56)	1.74	31	0.04

**Table 3:** Means and standard deviations for the maternal study variables.

Groups	Time		BDI	FCV-19S	PAI	PPBQ- Impaired Attachment	PSI	IES-R
EG	T0	M SD	8.10 6.17	13.8 6.73	65.5 10.6			
		Skewness Kurtosis	1.09 0.88	0.98 −0.11	−0.38 −0.93			
	T1	M SD	4.05 4.43	13.8 6.73		1.23 1.51	54.3 8.56	0.31 0.42
		Skewness Kurtosis	0.56 −1.21	0.98 −0.11		1.29 1.24	1.56 3.11	1.37 −0.13
	T2	M SD	3.88 4.25	10.9 4.02		0.90 1.03	53.1 6.14	0.47 0.40
		Skewness Kurtosis	0.54 −1.34	0.73 −0.83		0.79 −0.60	1.48 2.11	1.56 0.45
CG	T0	M SD	5.30 4.79	13.5 7.51	61.7 10.8			
		Skewness Kurtosis	1.42 2.26	1.29 1.24	−0.05 −1.08			
	T1	M SD	5.89 4.70	14.8 8.32		2.16 2.21	63.9 4.32	0.22 0.51
		Skewness Kurtosis	1.56 2.89	1.08 0.14		−1.36 1.29	−0.43 1.94	1.32 0.82
	T2	M SD	5.95 4.23	12.9 5.71		1.98 1.90	63.7 4.44	0.20 0.73
		Skewness Kurtosis	1.34 2.29	1.08 0.14		0.54 −0.60	0.03 −0.50	1.20 0.00

**Table 4:** Means and standard deviations for the paternal study variables.

Groups	Time		BDI	FCV-19S	PAAS- Global Attachment	PSI	IES-R
EG	T0	M SD	3.26 3.19	14.9 7.31	64 6.66		
		Skewness Kurtosis	0.40 −1.67	0.84 0.25	−0.03 0.25		
	T1	M SD	3.05 2.90	14.8 7.34		49.7 7.92	0.38 0.39
		Skewness Kurtosis	0.38 −1.55	1.49 1.88		1.06 0.67	1.47 0.67
	T2	M SD	2.79 2.66	12.7 4.45		50.1 8.22	0.37 0.38
		Skewness Kurtosis	0.51 −1.19	0.99 0.41		0.88 0.16	1.60 0.16
CG	T0	M SD	5.71 5.66	15.0 6.23	68.4 7.62		
		Skewness Kurtosis	0.83 −0.23	0.69 −1.51	0.81 −0.12		
	T1	M SD	6.57 6.66	20.8 9.37		70.3 3.58	0.82 0.44
		Skewness Kurtosis	1.40 1.56	0.11 −1.60		0.12 −0.44	0.88 −0.44
	T2	M SD	7.29 6.06	16.9 5.90		66.6 2.73	0.82 0.28
		Skewness Kurtosis	1.50 2.43	0.08 −1.67		−0.00 −0.87	0.86 −0.87

## Data Availability

The data presented in this study are available on request from the corresponding author.
